# S14G-humanin alleviates acute lung injury by inhibiting the activation of NF-κB

**DOI:** 10.18632/aging.205267

**Published:** 2023-12-04

**Authors:** Yunlong Wu, Hui Zhang, Lingbo Guan, Xiangli Jia, Mei Wang

**Affiliations:** 1 Department of ICU, The First People’s Hospital of Linping District, Hangzhou 311100, China

**Keywords:** S14G-humanin, acute lung injury, NLRP3, oxidative stress, NF-κB

## Abstract

Acute lung injury (ALI) is characterized by severely damaged alveoli and blood vessels, seriously affecting the health of patients and causing a high mortality rate. The pathogenesis of ALI is complex, with inflammatory reactions and oxidative stress (OS) mainly involved. S14G humanin (HNG) is derived from humanin (HN), which is claimed with promising anti-inflammatory functions. Herein, the protective influence of HNG on ALI will be explored in a mouse model. The ALI model was established in mice via intratracheal instillation of 3 mg/kg LPS, followed by an intraperitoneal injection of 3 and 6 mg/kg HNG, respectively. Thicker alveolar walls, aggravated neutrophil infiltration, and increased wet weight/dry weight (W/D) ratio were observed in ALI mice, accompanied by an aggravated apoptotic state, all of which were notably alleviated by HNG. Furthermore, increased number of total cells and neutrophils in bronchoalveolar lavage fluid (BALF), elevated secretion of inflammatory cytokines, enhanced reactive oxygen species (ROS) and Malondialdehyde (MDA) levels, and declined superoxide dismutase-2 (SOD2) levels were observed in ALI mice, which were markedly ameliorated by HNG. Moreover, the upregulated levels of NOD-like receptor family pyrin domain containing 3 (NLRP3), caspase-1, and caspases cleave gasdermin D N/caspases cleave gasdermin D FL (GSDMD N/GSDMD FL) in ALI mice were signally repressed by HNG. Lastly, the upregulation of Toll-like receptor 4 (TLR4) and p-p65/p65, and downregulation of IκB-α observed in ALI mice were sharply reversed by HNG. Collectively, HNG alleviated the ALI in mice by inhibiting the activation of nuclear factor kappa B (NF-κB) signaling.

## INTRODUCTION

Acute lung injury (ALI) is a disease state in which the normal structures of alveoli and blood vessels are severely damaged, with diffuse edema in lung tissue as the main pathological change [1, 2]. Dyspnea is gradually increased, leading to the development of hypoxemia that is difficult to resolve, even with high oxygen concentrations. When the disease progresses to an oxygenation index < 200, acute respiratory distress syndrome (ARDS) develops [3, 4]. At present, a common view is that ALI and ARDS are two stages of the same disease development process. According to statistics, about 55% of ALI instances will aggravate within 3 days and develop into ARDS [5]. The existing treatment methods for ALI/ARDS are limited. The core treatment method is mechanical ventilation using positive end-expiratory pressure (PEEP). Furthermore, prone position ventilation technology and high-frequency oscillation ventilation technology (HFOV) are combined, along with anti-inflammatory therapy, immunotherapy, lung recruitment ventilation, extracorporeal membrane oxygenator (ECMO), and the use of statins [6, 7]. However, the treatment cost is high and its effectiveness is rarely satisfactory [8]. According to the statistics of the United States Health Department, about 200,000 cases of ARDS and nearly 75,000 resulting deaths are reported in China annually. Nearly 3 million ALI/ARDS patients are diagnosed worldwide every year, with about 10% of all being severe cases. 24% of ALI/ARDS patients are treated with mechanical ventilation, and the mortality rate is between 35% and 46% [9].

When suffering from severe infection, or increased intestinal mucosal permeability caused by various injury factors such as alcohol consumption and trauma, sepsis develops due to increased LPS exposure. Excessive LPS enters the body and activates macrophages by binding to toll-like receptor 4 (TLR4) on the cell surface, which then activates the nuclear factor kappa-B (NF-κB) pathway to upregulate the expression of inflammatory factors, promoting the aggravation of inflammatory injury. The lung parenchyma and blood vessels are damaged by reactive oxygen species (ROS), elastase, and other substances, which contribute to the development of ALI/ARDS [10]. Controlling the enriched inflammation and ROS-induced OS could be promising methods for treating ALI.

HN, originally discovered in patients with Alzheimer’s disease, is a 24-nucleotide polypeptide encoded by an open reading frame (ORF) within the gene of the 16S ribosomal subunit in the mitochondrial genome with neuroprotective effects [11]. HNG is a humanin derivative that replaces the original valine at the 14th position with glutamic acid, it has high biological activity at nanomolar concentrations. HNG has been widely applied in animal and cell experiments [12, 13]. Recently, HNG is claimed with prominent anti-inflammatory properties. In UV-B-treated retinal endothelial cells, the NLRP3 inflammasome was inhibited by HNG to exert a protective effect [14]. In LPS-stimulated human dental pulp cells, the inflammation was repressed by HNG via TLR4/MyD88/NF-κB signaling [15]. Herein, we examined whether HNG possesses a beneficial effect against inflammatory response in an ALI mice model and explored the underlying mechanism, aiming to find a novel approach for the treatment of lung injury disease.

## RESULTS

### HNG alleviated the pathological changes in lung tissues of ALI mice

**Figure 1:**
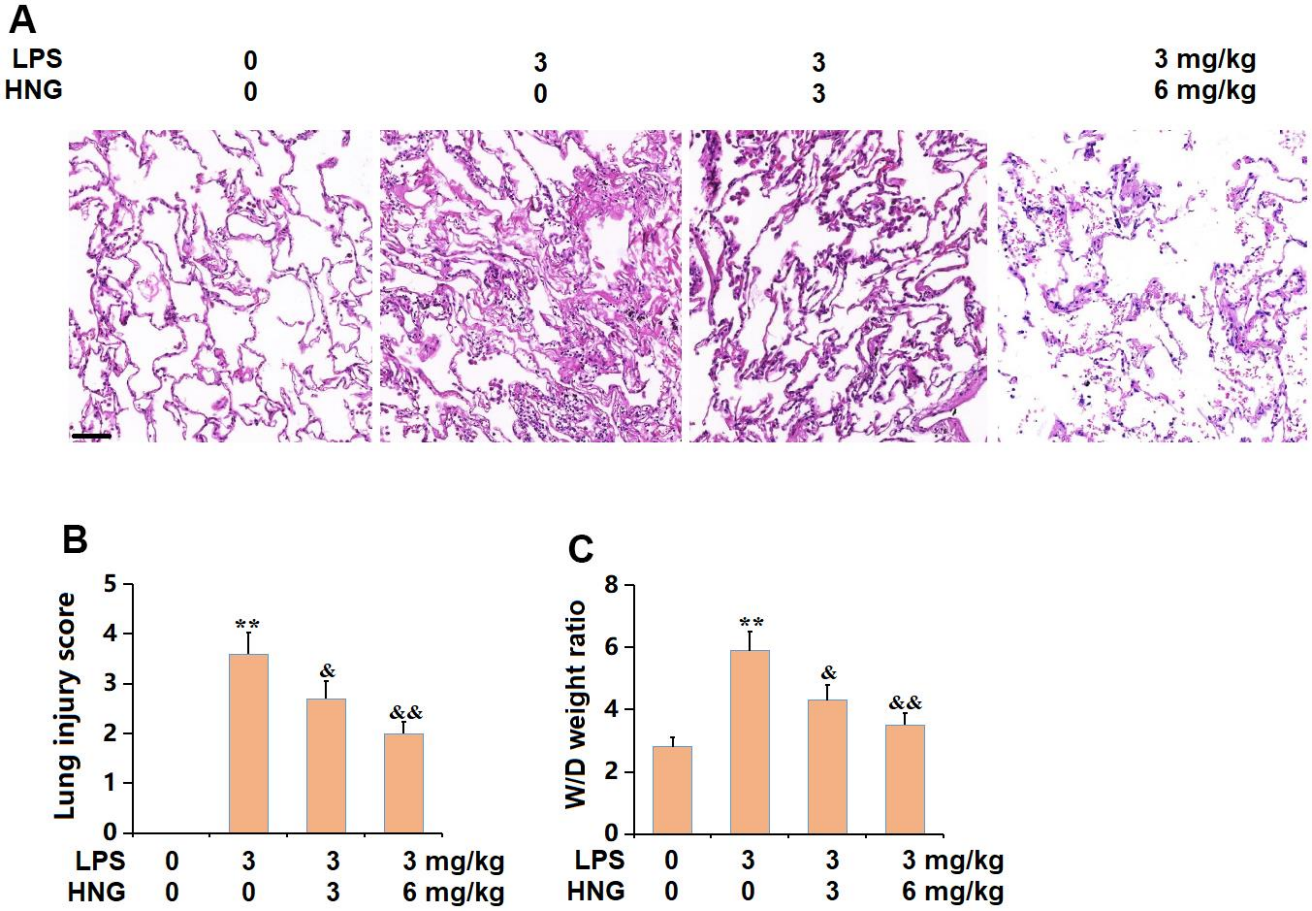
**S14G humanin (HNG) alleviated the pathological changes in lung tissues of acute lung injury (ALI) mice.** (**A**) Representative images of HE staining on lung tissues. Scale bar, 250 μm; (**B**) Lung injury score; (**C**) W/D weight ratio (n=6, **, P<0.05 vs. Control; &, P<0.05, 0.01 vs. LPS group).

The pathological state in lung tissues was checked using the HE staining assay, images of which are shown in Figure 1A. In the control group, the surface was smooth, the alveoli were regular under the microscope, and the cell morphology was normal. In the LPS group, scattered red spots were observed on the lung surface, and the alveolar wall was significantly thicker than that of the control group, with a large number of neutrophils infiltrating and red blood cells infiltration in the alveolar cavity. In the 3 mg/kg HNG and 6 mg/kg HNG groups, the inflammatory pathological changes to the lung tissue were significantly alleviated, and the infiltration of neutrophils and other inflammatory cells was significantly reduced. The markedly increased lung injury score in the LPS group was signally repressed by 3 mg/kg and 6 mg/kg HNG (Figure 1B). Moreover, the W/D weight ratio in the control, LPS, 3 mg/kg HNG, and 6 mg/kg HNG groups was 2.8, 5.9, 4.3, and 3.5, respectively (Figure 1C). The pathological symptoms in ALI mice were promisingly ameliorated.

### HNG repressed the infiltration of inflammatory cells in the BALF of ALI mice

**Figure 2:**
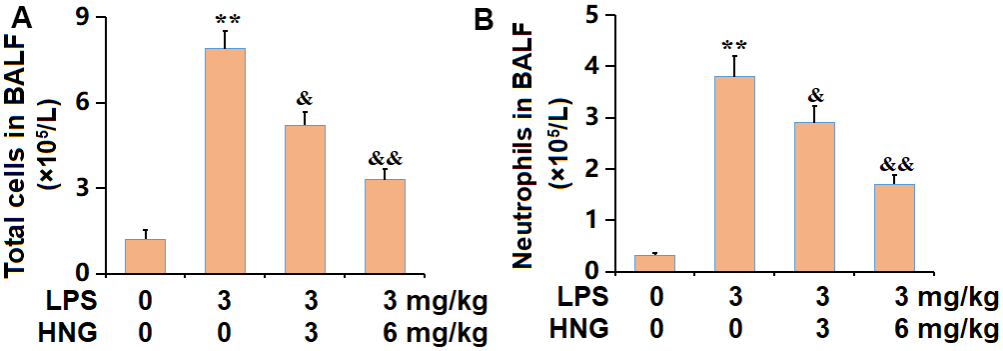
**S14G humanin (HNG) repressed the infiltration of inflammatory cells in the BALF of ALI mice.** (**A**) Total cells in BALF (×10^5^/L). (**B**) Neutrophils in BALF (×10^5^/L) (n=6, **, P<0.05 vs. Control; &, P<0.05, 0.01 vs. LPS group).

To confirm the repressed inflammation in lung tissues, BALF was collected from each animal. The number of total cells in BALF was notably increased from 1.2 to 7.9 ×10^5^/L, which was markedly reduced to 5.2 and 3.3 ×10^5^/L by 3 mg/kg and 6 mg/kg HNG, respectively (Figure 2A). Furthermore, the number of neutrophils in the control, LPS, 3 mg/kg HNG, and 6 mg/kg HNG groups was 0.32, 3.8, 2.9, and 1.7 ×10^5^/L, respectively (Figure 2B). A repressive effect of HNG on the infiltration of inflammatory cells in the BALF of ALI mice was observed.

### HNG ameliorated the inflammation in lung tissues of ALI mice

**Figure 3:**
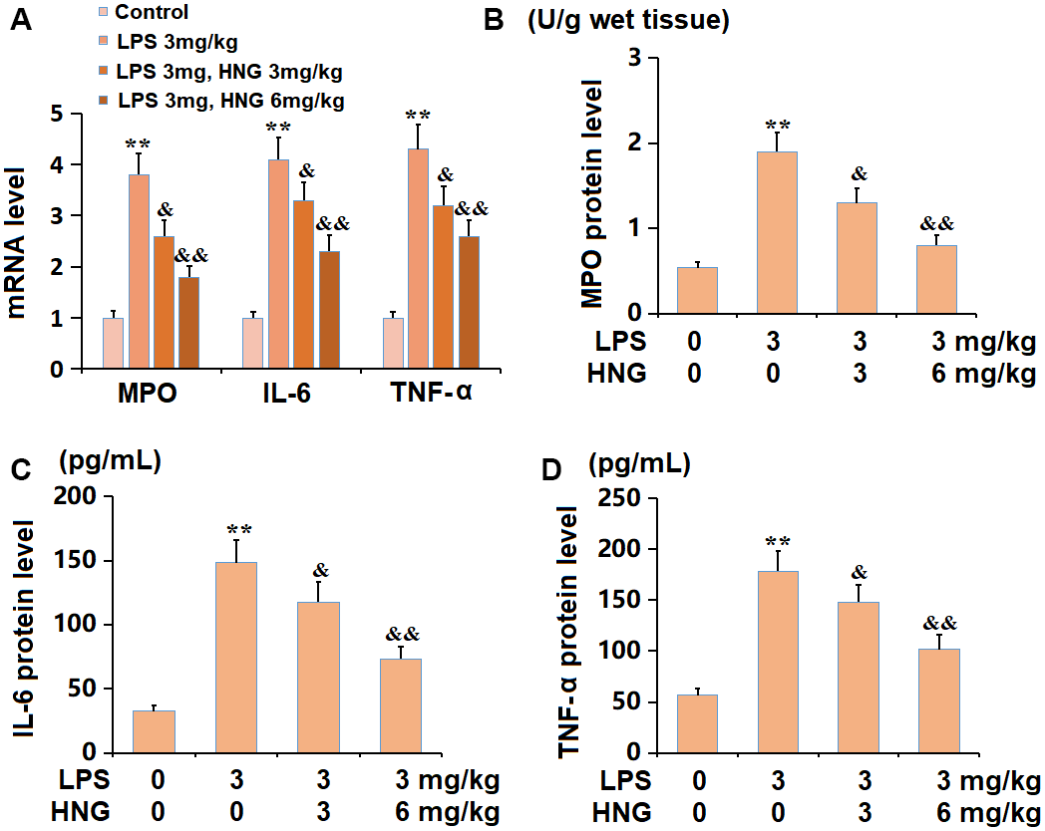
**S14G humanin (HNG) ameliorated the inflammation in lung tissues of ALI mice.** (**A**) mRNA level of MPO, mRNA level of IL-6, and mRNA level of TNF-α. (**B**) The protein level of MPO. (**C**) The protein level of IL-6. (**D**) The protein level of TNF-α (n=6, **, P<0.05 vs. Control; &, P<0.05, 0.01 vs. LPS group).

The mRNA levels of myeloperoxidase (MPO), interleukin 6 (IL-6), and tumour necrosis factor alpha (TNF-α) were signally increased in the LPS group, but notably reduced by 3 mg/kg and 6 mg/kg HNG (Figure 3A). Furthermore, the protein level of MPO in ALI mice was elevated from 0.54 to 1.9 U/g wet tissue, which was markedly repressed to 1.3 and 0.8 U/g wet tissue by 3 mg/kg and 6 mg/kg HNG, respectively. The IL-6 levels in the control, LPS, 3 mg/kg HNG, and 6 mg/kg HNG groups were 56.9, 178.4, 148.2, and 101.5 pg/mL, respectively. Moreover, the TNF-α level in ALI mice was increased from 32.6 to 148.2 pg/mL, which was dramatically reduced to 117.6 and 73.4 pg/mL by 3 mg/kg and 6 mg/kg HNG, respectively (Figure 3B).

### HNG repressed the OS in lung tissues of ALI mice

**Figure 4:**
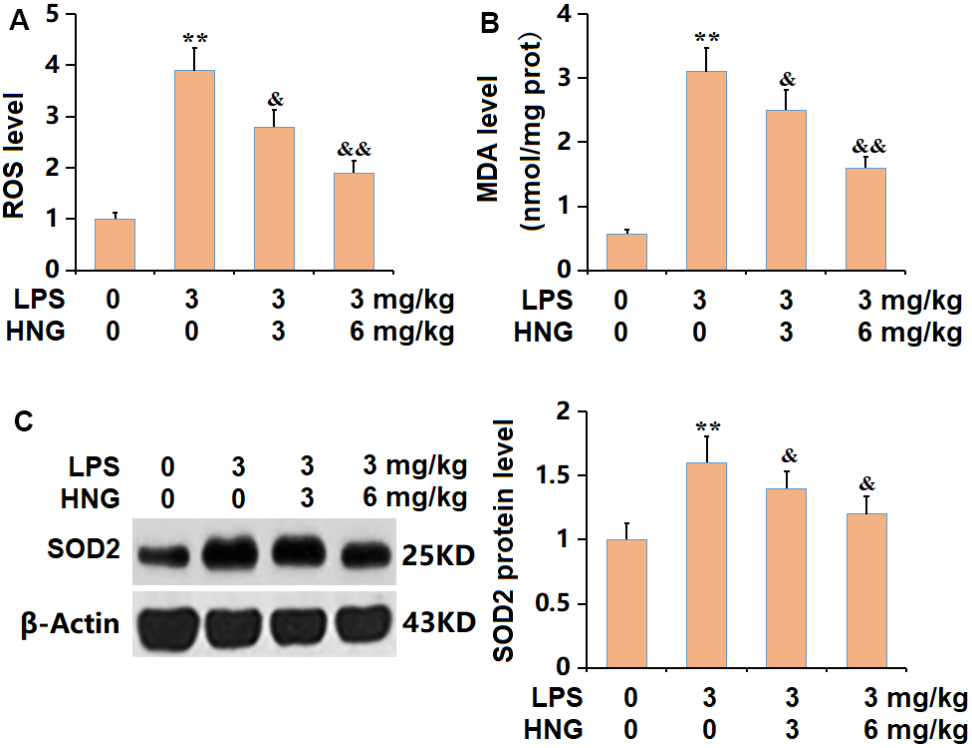
**S14G humanin (HNG) repressed the oxidative stress in lung tissues of ALI mice.** (**A**) ROS level. (**B**) MDA level. (**C**) SOD2 protein level (n=6, **, P<0.05 vs. Control; &, P<0.05, 0.01 vs. LPS group).

The largely increased ROS level observed in ALI mice was signally inhibited by 3 mg/kg and 6 mg/kg HNG (Figure 4A). The MDA level was observably increased from 0.57 to 3.1 nmol/mg prot in the LPS group, then markedly suppressed to 2.5 and 1.6 nmol/mg prot by 3 mg/kg and 6 mg/kg HNG, respectively (Figure 4B). Furthermore, the declined SOD2 level in ALI mice was sharply increased by 3 mg/kg and 6 mg/kg HNG (Figure 4C). A suppressive property of HNG against OS was observed in ALI mice.

### HNG alleviated the apoptosis in lung tissues of ALI mice

**Figure 5:**
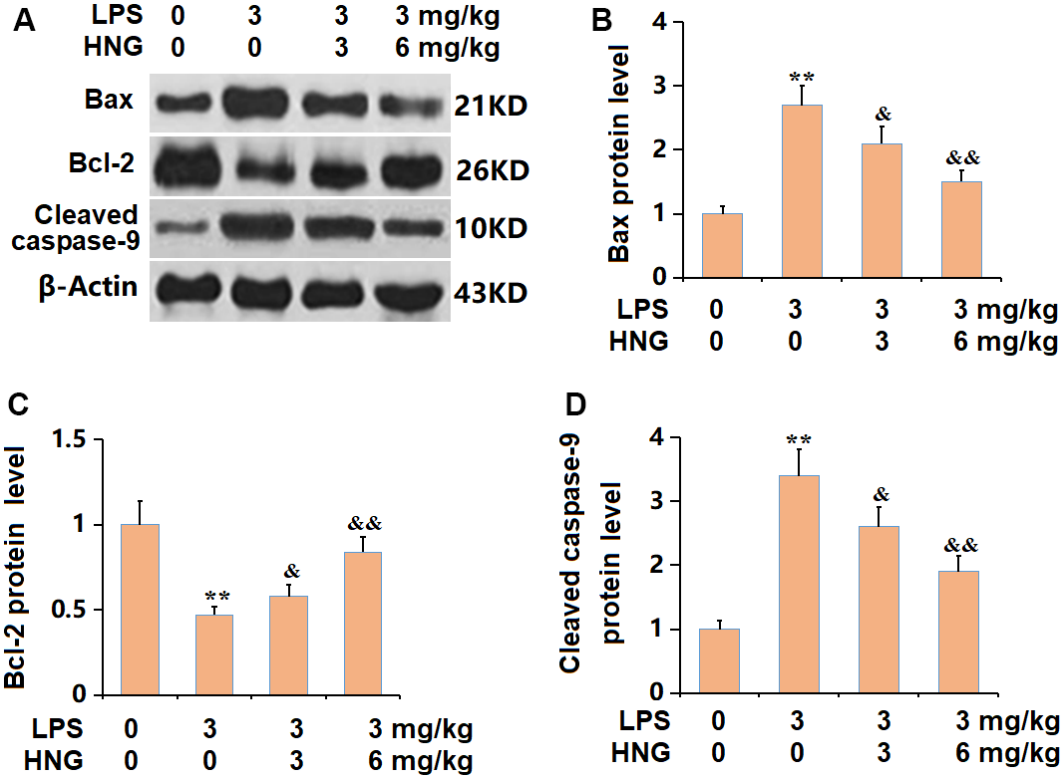
**S14G humanin (HNG) alleviated the apoptosis in lung tissues of ALI mice.** (**A**) The protein level of apoptosis related genes was determined by western blots. (**B**) Analysis of protein level of Bax level. (**C**) Analysis of protein level of Bcl-2 level. (**D**) Analysis of protein level of cleaved caspase-9 level (n=6, **, P<0.05 vs. Control; &, P<0.05, 0.01 vs. LPS group).

Subsequently, the apoptotic state in lung tissues was determined. The Bax and caspase-9 level was found signally increased, while the Bcl-2 level was markedly reduced in the LPS group, all of which were sharply reversed by 3 mg/kg and 6 mg/kg HNG (Figure 5A–5D), suggesting an anti-apoptosis property of HNG in ALI mice.

### HNG suppressed the activation of NLRP3 signaling in lung tissues of ALI mice

**Figure 6:**
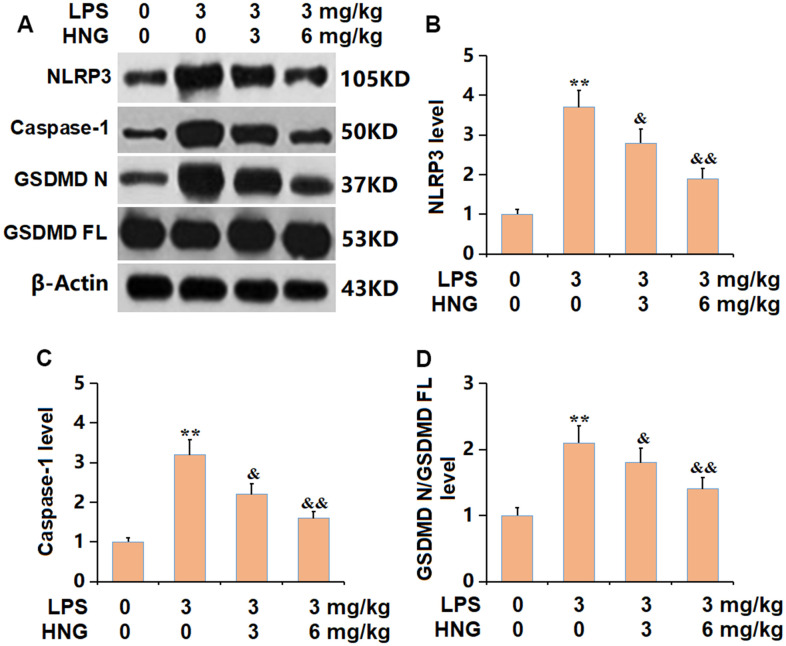
**S14G humanin (HNG) suppressed the activation of NLRP3 signaling in lung tissues of ALI mice.** (**A**) The protein level of genes related with activation of NLRP3 was determined by western blots. (**B**) Analysis of protein level of NLRP3 level. (**C**) Analysis of protein level of caspase-1 level. (**D**) Analysis of protein level of GSDMD N/GSDMD FL level (n=6, **, P<0.05 vs. Control; &, P<0.05, 0.01 vs. LPS group).

The NLRP3 inflammasome is reportedly activated in ALI [16]. The NLRP3, caspase-1, and GSDMD N/GSDMD FL levels in ALI mice were sharply increased, then signally reduced by 3 mg/kg and 6 mg/kg HNG (Figure 6A–6D), indicating a repressive effect of HNG on NLRP3 signaling in ALI mice.

### HNG inhibited TLR4/NF-κB signaling in lung tissues of ALI mice

**Figure 7:**
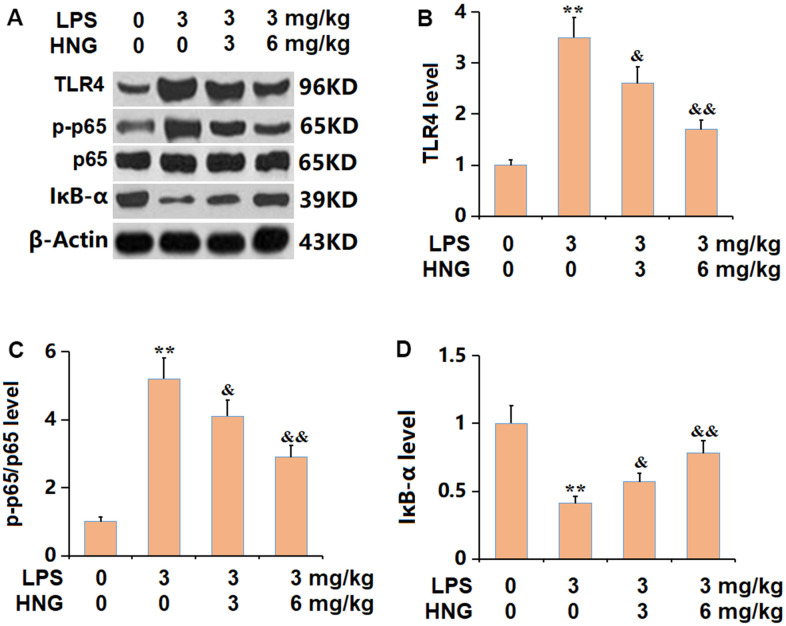
**S14G humanin (HNG) inhibited the TLR4/NF-κB signaling in lung tissues of ALI mice.** (**A**) The protein levels were detected using western blots. (**B**) Analysis of protein level of TLR4 level. (**C**) Analysis of protein level of p-p65/p65 level. (**D**) Analysis of protein level of IκB-α level (n=6, **, P<0.05 vs. Control; &, P<0.05, 0.01 vs. LPS group).

The TLR4/NF-κB axis is claimed to be responsible for the inflammatory activation in ALI [17]. The levels of TLR4 and p-p65/p65 were found signally increased, while the IκB-α level was markedly reduced in ALI mice, all of which were sharply reversed by 3 mg/kg and 6 mg/kg HNG (Figure 7A–7D), implying a suppressive effect of HNG on TLR4/NF-κB signaling in ALI mice.

## DISCUSSION

The pathogenesis of ALI is complex and has not been fully elucidated to date. However, OS is widely recognized as one of the main pathogeneses of ALI [18]. When the body produces a large amount of ROS under the action of various internal factors (such as ischemia, hypoxia, and inflammation) and external factors (such as stress and burns), ROS breaks the balance between the oxidation and antioxidant systems, resulting in cellular or tissue damages, the progression of which is named OS [19]. ROS are chemically active oxygen-containing atoms or groups produced in REDOX reactions, including superoxide anion (O_2_^-^), hydroxyl radical (-OH), hydrogen peroxide (H_2_O_2_), ozone (O_3_), and nitric oxide (NO). The strong oxidative function of these products contributes to serious damage to key components of cells such as DNA, lipids, and proteins. However, under physiological conditions, the endogenous defense system prevents the formation of ROS or removes these oxides, thereby protecting the body from oxidative damage [20]. Herein, consistent with previous studies [21, 22], a severe pathological change was observed in lung tissues of ALI mice, accompanied by aggravated apoptosis, which were markedly alleviated by HNG, suggesting its protective property against ALI in the animal model. Moreover, activated OS was observed in lung tissues of ALI mice, which was also claimed by Zhou [23] and Wang [24]. Following the administration of HNG, the OS state was signally ameliorated, implying that HNG might exert its anti-ALI function by repressing OS.

The NLRP3 inflammasome is an intracellular multi-protein complex containing NOD-like receptors, apoptosis-associated speck-like protein containing CARD (ASC), and caspase-1 [25]. It has been proven that the NLRP3 inflammasome participates in the development of inflammation [26]. Under inflammatory conditions, the NLRP3 inflammasome recognizes intracellular and extracellular danger signaling molecules (such as LPS) to induce a nonspecific immune response and recruit and activate pro-inflammatory protease caspase-1. The activated caspase-1 will further promote the maturation of interleukin 1β [27]. It is well known that cytokines interleukin-1β, tumor necrosis factor-α, and interleukin-6 are the most common pro-inflammatory mediators participating in the process of lung pathological injury, and are closely related to the severity of ALI [28]. Therefore, controlling the activation of the NLRP3 inflammasome may be effective treatment for ALI. Herein, severe inflammation, including enhanced infiltration of inflammatory cells in the BALF and increased release of cytokines in lung tissues, was observed in ALI mice, in line with Li’s report [29]. Following the administration of HNG, the inflammation state was markedly mitigated. Moreover, similar to the research conducted by Yang [30], NLRP3 inflammasome activation was observed in ALI mice, which was notably abolished by HNG, implying that the anti-inflammatory function of HNG might be correlated to the inhibition of the NLRP3 inflammasome.

NF-κB is a common transcription factor composed of P50, P52, P65, cRel and RelB, and it is confirmed that NF-κB regulates the expression of a variety of inflammatory factors, chemokines, and adhesion factors [31], which largely participate in the inflammatory response to ALI. In the quiescent state, the NF-κB protein binds to its inhibitor IκB, which inactivates NF-κB. When external stimuli such as inflammatory factors, cyclooxygenase (COX2), chemokines, adhesion factors, and colony-stimulating factors, bind to their corresponding receptors, NF-κB signaling is activated [32]. Studies have found that ALI development is closely correlated to the activation of NF-κB signaling, and inhibiting the NF-κB pathway is found to effectively alleviate the symptoms of ALI [33, 34]. Herein, NF-κB signaling was found markedly activated in ALI mice, in line with previous studies [35, 36]. The protection by HNG against ALI symptoms was along with an inhibition of NF-κB activity, implying that the anti-ALI function of HNG might be correlated to the suppression of NF-κB signaling.

## CONCLUSIONS

Taken together, our study indicates that HNG alleviated ALI in mice by inhibiting the activation of the NF-κB pathway. Our findings shed a light on further investigations into the treatment of ALI.

## MATERIALS AND METHODS

### Animals and treatments

All animal experiments were performed following the ARRIVE guidelines (http://www.nc3rs.org.uk/arrive-guidelines). C57BL/6 male mice were obtained from Yishang Biotech (Shanghai, China) and bred in individually ventilated cages in an Animal Care Facility with sufficient food and water at 12-hour light-dark cycle, 21° C, 35% humidity condition for a week. Then the mice were divided into 4 groups (n=6/group): Control, LPS, 3 mg/kg HNG, and 6 mg/kg HNG. The ALI model was established in mice by intratracheal instillation of 3 mg/kg LPS, followed by an intraperitoneal injection of 3 and 6 mg/kg HNG for 24 hours. This study is approved by the Ethics Committee and Animal Care Committee of The First People’s Hospital of Linping District.

### Hematoxylin and eosin (H&E) staining

The obtained lung tissue samples were placed in 4% formaldehyde solution for 48 hours. Gradient dehydration was performed with 70%, 80%, 90%, 95%, and 100% alcohol, respectively. Specimens were made transparent twice with xylene, and placed in dissolved paraffin, and kept in a wax solubilizing box for 2 hours at 65° C. Paraffin was observed to be completely immersed in the tissue and then embedded. After cooling and solidification, paraffin blocks of specimen tissue were automatically cut in a transverse section in the middle with a microtome at a thickness of 4 μm. Slices were scalded in hot water and then reattached to glass slides and kept in an oven at 58° C for 4 h. After deparaffinization and washing with water, slides were stained with hematoxylin for 3 min, then differentiated with 1% hydrochloric acid alcohol for 20 s, and finally stained with eosin for 5 min. Dehydration was carried out with 70%, 80%, 90%, 95%, and 100% alcohol in turn, and each was kept for 3 min. Transparency was carried out with xylene and kept for 5 min. Finally, neutral resin glue was used to complete the sealing.

### Pulmonary W/D weight ratio

After the mice were sacrificed, the right middle lobe of the lung was removed, and the surface water was sucked with water-absorbent paper. The wet weight (W) was recorded by electronic balance. Then tissues were placed in a drying oven at 60° C, and drying was stopped when the weight did not change. The tissues were weighed again and recorded as dry weight (D). W/D value can be used to evaluate pulmonary edema.

### Counting of total cells and neutrophils in BALF

Mice were anesthetized with 35 mg/kg 1% sodium pentobarbital, and BALF samples were collected. Then animals were sacrificed and the trachea was separated under sterile conditions, which was intubated to the left main bronchus and ligated. Samples were centrifuged at 3000 r/min for 10 min and the supernatant was frozen and stored. Cells obtained by centrifugation were stained using the Wright Giemsa method, and the number of total cells and neutrophils was observed and recorded by a microscope (Leica, Wetzlar, Germany).

### Enzyme-linked immunosorbent assay (ELISA)

The secretion of Myeloperoxidase (MPO), Interleukin 6 (IL-6), and Tumour Necrosis Factor-alpha (TNF-α) was detected with a commercial ELISA kit (Cat#EM0010, EM0121, EM0183, FineTest, Wuhan, China). The lung tissue of each animal was collected and the homogenate was achieved, followed by collecting the supernatant to be diluted at a 1:1 ratio. After loading samples into wells, 50 μL Biotin-labeled antibody was added to be cultured for 60 min, followed by loading 80 μL horseradish peroxidase (HRP)-loaded secondary antibody. After 10 min incubation at 37° C, 50 μL TMB substrates were added and cultured at 37° C for 10 min. After loading with 50 μL stop solution, the optical density (OD) value was detected at 450nm utilizing a microplate reader (MD, USA).

### MDA and glutathione peroxidase (GSH-PX) level detection

The MDA level in lung tissues was detected using the thiobarbituric acid (TBA) method with a commercial kit (Qingdao Jisskang Biotechnology, Qingdao, China). The instruction of the kit was strictly followed.

### Dichloro-dihydro-fluorescein diacetate (DCFH-DA) assay

The lung tissue was cut into pieces and loaded with the PBS buffer, followed by centrifugation and discarding the supernatant. The tissue suspension was obtained after resuspending using the PBS buffer. DCFH-DA (Cat#S0033M, Beyotime, Beijing, China) was diluted in serum-free medium (dilution ratio 1: 1000), and the final concentration of DCFH-DA was 10 μmol/L. DCFH-DA was added to the tissue suspension, and incubated at room temperature in a dark place for 20 min. The tissue suspension was washed with serum-free medium, and the fluorescence intensity of intracellular ROS was detected by a fluorescent microplate reader (Thermo Fisher Scientific, USA) at 488 and 525 nm of maximum excitation and emission spectra, respectively.

### Real-time polymerase chain reaction (RT-PCR)

Lung tissues were collected to extract total RNAs according to TRIzol reagent instructions (CWBIO, Beijing, China). Subsequently, cDNA synthesis was performed utilizing the RT-PCR reverse transcription kit (QIAGEN, USA), followed by conducting the PCR amplification in the PCR instrument (Thermo Fisher Scientific, USA). The internal reference gene was Glyceraldehyde-3-phosphate dehydrogenase (GAPDH) and the gene level were determined utilizing the 2^−ΔΔCt^ method. The following primers were used in the study: (mouse) MPO forward: 5’-GACAT GCCCA CCGAA TGACAA-3’, (mouse) MPO reverse: 5’-CAGGCAACCAGCGTACAAAG-3’; (mouse) TNF-α forward: 5’-CAGAGGGAAGAGTTCCCCAG-3’, (mouse) TNF-α reverse: 5’-CCTTGGTCTGGTAGGAGACG-3’; (mouse) IL-6 forward: 5’-CACCGGGAAC GAAAGAGAAG-3’, (mouse) IL-6 reverse: 5’-TCTGA GGTGCCCATGCTACA T-3’; (mouse) GAPDH forward: 5’-TGACCTCAACTACATGGTCTACA-3’, (mouse) GAPDH reverse: 5’-CTTCCCATTCTCGGCCTTG-3’.

### Western blotting assay

Lung tissues were collected to extract total proteins to be quantified with the bicinchoninic acid (BCA) method (Elabscience, USA). The separation of proteins was conducted utilizing a 12% sodium dodecyl sulfate (SDS)-polyacrylamide gel (PAGE), followed by transferring the separated proteins onto the polyvinylidene fluoride (PVDF) membrane. The blocking was conducted using the 5% skim milk and primary antibodies against SOD2 (1:1000, Cat#sc-137254, Santa Cruz Biotechnology, USA), Bax (1:2000, Cat#AF820, R&D System, USA), Bcl-2 (1: 1000, Cat#AB112, Beyotime Biotechnology, China), cleaved caspase-9 (1:800, Cat#AF5244, Affinity, China), NLRP3 (1:1000, Cat#AF6555, Affinity, China), caspase-1 (1:1000, Cat#2225, CST, USA), GSDMD N (1:800, Cat#DF12275, China), GSDMD FL (1:800, Cat#AF4012, Affinity, China), TLR4 (1:1000, Cat#AF7017, Affinity, China), p-p65 (1:800, Cat#sc-101752, Santa Cruz Biotechnology, USA), p65 (1:2000, Cat#DF7003, Affinity, China), p-IκB-α (1:1000, Cat#sc-520548, Santa Cruz Biotechnology, USA), IκB-α (1:2000, Cat#sc-520432, Santa Cruz Biotechnology, USA), and β-actin (1:2000, #4967, CST, USA). Subsequently, the secondary antibody (1:2000, Servicebio, China) was introduced and cultured for 60 min. The ECL solution was loaded for exposure and the protein level was quantified with the software Image J. Briefly, bands were scanned and the background was subtracted. Integrated optical density was then calculated to index the protein level.

### Statistical analysis

Mean ± Standard Deviation (SD) was expressed and the one-way analysis of variance method was utilized with a Scheffe test as the post-hoc test for analysis with the GraphPad Prism software 6.0. P < 0.05 was considered a statistically significant difference.

### Data availability statement

The data are available upon reasonable request from the corresponding author.
